# Brain regions preferentially responding to transient and iso-intense painful or tactile stimuli

**DOI:** 10.1016/j.neuroimage.2019.01.039

**Published:** 2019-05-15

**Authors:** Q. Su, W. Qin, Q.Q. Yang, C.S. Yu, T.Y. Qian, A. Mouraux, G.D. Iannetti, M. Liang

**Affiliations:** a School of Medical Imaging and Tianjin Key Laboratory of Functional Imaging, Tianjin Medical University, Tianjin, China; b Department of Radiology and Tianjin Key Laboratory of Functional Imaging, Tianjin Medical University General Hospital, Tianjin, China; c CAS Center for Excellence in Brain Science and Intelligence Technology, Chinese Academy of Sciences, Shanghai, China; d MR Collaboration, Siemens Healthcare Northeast Asia, Beijing, China; e Institute of Neuroscience (IoNS), Université Catholique de Louvain, Brussels, Belgium; f Neuroscience and Behaviour Laboratory, Istituto Italiano di Tecnologia, Rome, Italy; g Department of Neuroscience, Physiology and Pharmacology, University College London, London, UK

## Abstract

How pain emerges from cortical activities remains an unresolved question in pain neuroscience. A first step toward addressing this question consists in identifying brain activities that occur preferentially in response to painful stimuli in comparison to non-painful stimuli. A key confound that has affected this important comparison in many previous studies is the intensity of the stimuli generating painful and non-painful sensations. Here, we compared the brain activity during iso-intense painful and tactile sensations sampled by functional MRI in 51 healthy participants. Specifically, the perceived intensity was recorded for every stimulus and only the stimuli with rigorously matched perceived intensity were selected and compared between painful and tactile conditions. We found that all brain areas activated by painful stimuli were also activated by tactile stimuli, and vice versa. Neural responses in these areas were correlated with the perceived stimulus intensity, regardless of stimulus modality. More importantly, among these activated areas, we further identified a number of brain regions showing stronger responses to painful stimuli than to tactile stimuli when perceived intensity was carefully matched, including the bilateral opercular cortex, the left supplementary motor area and the right frontal middle and inferior areas. Among these areas, the right frontal middle area still responded more strongly to painful stimuli even when painful stimuli were perceived less intense than tactile stimuli, whereas in this condition other regions showed stronger responses to tactile stimuli. In contrast, the left postcentral gyrus, the visual cortex, the right parietal inferior gyrus, the left parietal superior gyrus and the right cerebellum had stronger responses to tactile stimuli than to painful stimuli when perceived intensity was matched. When tactile stimuli were perceived less intense than painful stimuli, the left postcentral gyrus and the right parietal inferior gyrus still responded more strongly to tactile stimuli while other regions now showed similar responses to painful and tactile stimuli. These results suggest that different brain areas may be engaged differentially when processing painful and tactile information, although their neural activities are not exclusively dedicated to encoding information of only one modality but are strongly determined by perceived stimulus intensity regardless of stimulus modality.

## Highlights

• Transient painful and tactile stimuli activate the same brain areas.
• Neural activity in these areas encode stimulus intensity.
• Among these areas, a few may be engaged differentially in pain and touch processing.

## 1 Introduction

Transient nociceptive stimuli causing pain elicit robust responses in a set of widely distributed brain regions including the thalamus, the primary and secondary somatosensory areas, the insula, the cingulate cortex and also some areas in the frontal and parietal lobes (Boly et al., 2008; Garcia-Larrea and Peyron, 2013; Iannetti et al., 2005b; Ingvar, 1999; Jones, 1998; Ploghaus et al., 1999; Stern et al., 2006; Talbot et al., 1991; Tracey and Mantyh, 2007; Wager et al., 2013; Whyte, 2008). Many studies explicitly suggest that pain perception is consequent to the neural activity of these brain areas (Apkarian et al., 2005; Brodersen et al., 2012; Duerden and Albanese, 2013; Kuo et al., 2017; Moisset and Bouhassira, 2007; Rainville, 2002). However, none of these brain areas is exclusively involved in nociceptive processing as they are all also activated by non-nociceptive sensory stimuli that do not cause painful percepts (Mouraux et al., 2011), and even in pain-free patients (Salomons et al., 2016) (for a recent review on the topic, see Mouraux and Iannetti, 2018). This evidence indicates that the function of these regions is largely unrelated to pain perception, but is instead related to the detection of sudden environmental events that require immediate attention, regardless of the sensory channel through which these events are conveyed (Downar et al., 2000, 2001; 2002; Iannetti and Mouraux, 2010, 2015; Legrain et al., 2011; Novembre et al., 2018). These two interpretations are not mutually exclusive. Rather, they probably reflect different facets of the complex functions served by these brain regions. In fact, many studies have attempted to identify the neural correlates of pain using a variety of brain imaging techniques and suggested neural activities that might be preferentially involved in pain processing. For example, it has been claimed, on the basis of recordings using intracerebral local field potentials (LEPs), scalp electroencephalography (EEG), functional magnetic resonance imaging (fMRI) and positron emission tomography (PET), that the secondary somatosensory cortex (S2) (Peyron et al., 2002), the insula (both posterior (Isnard et al., 2011; Peyron et al., 2002) and anterior (Peyron et al., 2002)) and the anterior cingulate cortex (ACC) (Lieberman and Eisenberger, 2015) might contain neural activities selective to pain. However, in these studies an important confound was neglected: when comparing brain responses to painful stimuli with those to non-painful stimuli stimulus intensity was not matched. Indeed, given that the amplitude of neural activity in many brain areas was found to correlate with stimulus intensity (Coghill et al., 1999; Iannetti et al., 2008), it remains unclear whether these previously identified brain areas responded to pain preferentially or simply because painful stimuli were more intense (Hu and Iannetti, 2016).

Therefore, in the present study, we first formally tested whether the amplitude of neural activity in the brain areas responding to painful and tactile stimuli correlated with perceived stimulus intensity. This first test proves the necessity of matching perceived intensity when comparing brain responses to painful nociceptive stimuli and brain responses to non-painful tactile stimuli. We then performed such comparison, using carefully matched painful and tactile stimuli, to test whether there were brain regions preferentially responding to painful stimuli than to tactile stimuli, ruling out the possibility that differences in fMRI responses evoked by painful and tactile stimuli were due to difference in their perceived intensity. Finally, we further tested whether modality preference of the identified brain regions could still be detected when the perceived stimulus intensity of the preferred modality was lower than that of the non-preferred modality.

## 2 Materials and methods

### 2.1 Participants

51 healthy young adults (mean age: 24 ± 2.29 years; 34 females) were recruited through college and community advertisements and paid for their participation. All participants were Chinese and right-handed. Participants were carefully screened to ensure that they had no history of brain injuries, pain disorders, any psychiatric or neurological diseases, alcohol abuse, drug abuse, or hypertension, and that they had no contraindications to MRI examination.

All participants provided written informed consent prior to the experiment, and the experimental procedures were approved by the Medical Research Ethics Committee of Tianjin Medical University General Hospital.

### 2.2 Experimental design

While lying in the scanner, participants received stimuli of two sensory modalities (painful and tactile) and two stimulus physical intensities (low and high). Painful stimuli were laser pulses delivered on the right foot dorsum within the sensory territory of the superficial peroneal nerve, and tactile (i.e., non-painful) stimuli were transcutaneous electrical pulses delivered over the superficial peroneal nerve of the right foot, similar to what were used in (Mouraux et al., 2011). The two levels of physical intensities (low and high) were determined for each type of stimulus (painful and tactile) for each individual participant before the scanning, using the following procedure: participants were first familiarized with a few laser stimuli; then a series of laser pulses of different energies was delivered, and participants were asked to rate the perceived intensity after each stimulus using a numerical rating scale (0 indicates no sensation and 10 indicates the worst pain imaginable); the physical intensities corresponding to the perceived intensity rating of 3 and 6 were used in the subsequent experiment during the fMRI scanning as the low and high painful stimulus intensities, respectively. This procedure was repeated for electrical stimuli to determine the low and high physical intensities for tactile stimuli (0 indicates no sensation and 10 indicates the strongest sensation as such electrical shock). The intensity of electrical stimuli was kept below the pain threshold in all participants to ensure that a non-painful, tactile sensation was elicited. Therefore, different physical intensities of both painful and non-painful stimuli were used in different participants to account for inter-subject variability in sensory sensitivity (Hu and Iannetti, 2019).

The experiment consisted of two sessions of fMRI data acquisition, with 24 trials organized in three ‘painful’ blocks and three ‘tactile’ blocks in each session (i.e., 4 trials in each block, 2 with high physical intensity and 2 with low physical intensity, randomly ordered). ‘Painful’ and ‘tactile’ blocks were presented in alternation, and their order was balanced across sessions and participants. In each trial a 10-s stimulation period was followed by a 10-s rating period. There was a 2-s interval between the onset of the trial and the onset of the stimulation period, and a 3-s interval between the end of the stimulation period and the beginning of the rating period. During the 10-s stimulation period, only one brief stimulus (either painful or tactile) was delivered at a random time (uniform distribution) for a jittering effect between trials. A white fixation cross was displayed at the center of the screen during the first 15-s period. During the rating period, a visual analogue scale (VAS, ranging from 0 to 10) (Hu et al., 2015; Steenbergen et al., 2012; Zhao et al., 2015, 2017) was presented on the screen and participants were instructed to rate the perceived intensity of the stimulus delivered in the same trial using a button box.

### 2.3 MRI data acquisition

MRI data of the study were acquired using a MAGNETOM Prisma 3T MR scanner (Siemens, Erlangen, Germany) with a 64-channel phase-array head-neck coil. Tight but comfortable foam padding was used to minimize head motion, and earplugs were used to reduce scanner noise. Blood-oxygenation level dependent (BOLD) signals were collected with a prototype simultaneous multi-slices gradient echo echo-planar imaging (EPI) sequence using the following parameters to achieve a good trade-off between spatial resolution and temporal resolution with a good signal-to-noise ratio at the same time: echo time (TE) = 30 ms, repetition time (TR) = 800 ms, field of view (FOV) = 222 mm × 222 mm, matrix = 74 × 74, in-plane resolution = 3 mm × 3 mm, flip angle (FA) = 54°, slice thickness = 3 mm, gap = 0 mm, number of slices = 48, slice orientation = transversal, bandwidth = 1690 Hz/Pixel, PAT (Parallel Acquisition Technique) mode, slice acceleration factor = 4, phase encoding acceleration factor = 2. A high-resolution 3D T1 structural image (two inversion contrast magnetization prepared rapid gradient echo sequence, MP2RAGE) was also acquired with the following parameters: TR/TE = 4000 ms/3.41 ms, inversion times (TI1/TI2) = 700 ms/2110 ms, FA1/FA2 = 4°/5°, matrix = 256 × 240, FOV = 256 mm × 240 mm, number of slices = 192, in-plane resolution = 1 mm × 1 mm, slice thickness = 1 mm, slice orientation = sagittal, total duration is 6 min 42 s.

### 2.4 Data preprocessing

The fMRI data were firstly preprocessed using Statistical Parametric Mapping (SPM8, http://www.ﬁl.ion.ucl.ac.uk/spm) with the following steps: realignment (correction for head motion-induced inter-volume displacement); normalized to the Montreal Neurological Institute (MNI) space using the unified normalization-segmentation procedure via the structural images; and spatially smoothed using a Gaussian kernel of 5-mm full-width at half-maximum (FWHM). The default high-pass temporal filtering (1/128 Hz cut-off) in SPM8 was applied to remove low-frequency noise and signal drifts from the fMRI time course of each voxel.

### 2.5 Matching perceived intensity between painful and tactile stimuli

To make sure that any detected difference in brain activity between painful and tactile conditions was not driven by differences in perceived stimulus intensity, a subset of painful and tactile trials with matched perceived intensity was selected using the following procedure: for a given laser stimulus with perceived intensity rating of *r*, all electrical stimuli with perceived intensity within the range of [*r*-0.5, *r*+0.5] were identified, and the electrical stimulus with the closest rating was selected to pair with that laser stimulus; if no electrical stimulus was identified within this range, the laser stimulus was labelled as unmatched. In this way, the selected pairs of the laser and electrical stimuli were matched on a trial-by-trial basis in terms of their perceived intensity. Note that, unless otherwise defined, the term “intensity-matched” or “iso-intense” in the present study refers to the fact that the *perceived* intensities were matched between painful and tactile stimuli.

#### 2.5.1 Analysis (1): Identification of brain areas where the neural activity correlate with perceived stimulus intensity regardless of stimulus modality

The rationale behind the necessity of matching perceived stimulus modality when comparing brain responses to painful and tactile stimuli is that the amplitude of such responses depends on the perceived intensity. To formally test this, we performed a general linear model (GLM) analysis to identify brain areas where the neural activity correlated with the perceived stimulus intensity regardless of stimulus modality. The two sessions were modeled as separate regressors and covariates in a single GLM. In the GLM, for each session the occurrence of all painful and tactile stimuli was collapsed into a single regressor with parametric modulation by their perceived intensity (i.e., stimulus subjective ratings), and the rating period was also modeled as an additional regressor. Six head motion parameters (estimated from the realignment step during fMRI data preprocessing) and the mean of each session were included as covariates in the GLM. The contrast maps corresponding to the subjective ratings of all stimuli in the first-level analysis were further entered into a second-level one-sample *t*-test to obtain group level results. A non-parametric permutation test (n = 5,000) corrected at cluster-level or voxel-level based on family-wise-error (FWE) method with a whole brain mask was used to determine the statistical significance (*P* < 0.05 corrected). This permutation and multiple correction procedure was performed using the software package SnPM13 (http://warwick.ac.uk/snpm).

#### 2.5.2 Analysis (2): General linear model of iso-intense painful vs. tactile stimuli

For each participant, first-level statistical parametric maps were obtained using a GLM with regressors modeling the stimulus occurrence of each of five event types (two sessions were modeled as separate regressors): intensity-matched painful stimuli, intensity-matched tactile stimuli, the remaining painful stimuli, the remaining tactile stimuli and the rating period. The temporal derivatives of the five conditions, the six head motion parameters and the mean of each session were also included in the GLM as covariates. Three contrast analyses were performed in each participant: (1) activation by intensity-matched painful stimuli, (2) activation by intensity-matched tactile stimuli, (3) differences in activation between intensity-matched painful and tactile stimuli. These individual contrast maps were fed into second-level analyses (one-sample *t*-test) to generate corresponding group-level results of the three contrast analyses. The statistical significance was then determined for each of the three group-level contrast results using the following methods.

As it has recently been shown that the GLM results are heavily dependent on the methods used for determining the statistical significance (Eklund et al., 2016), we reported four sets of results obtained using four different methods for correcting multiple comparisons problem. In this way we provide a systematic investigation of the GLM results and meanwhile evaluate the robustness of the results. The four sets of GLM results were obtained using statistical *P* values determined by different multiple comparisons correction methods: (1) non-parametric permutation test corrected at voxel-level using the software package SnPM13 (Results Set 1), (2) non-parametric permutation test corrected at cluster-level using SnPM13 (Results Set 2), (3) random field theory (RFT) corrected at voxel-level using the software package SPM8 (Results Set 3), (4) RFT corrected at cluster-level using SPM8 (Results Set 4). All the above correction methods were based on FWE method. For all sets of results, the statistical significance level was set to *P* < 0.05 after correction. For cluster-level corrections, the cluster-defining threshold was set to *P* < 0.001 before correction. For the non-parametric permutation test, we performed 5,000 permutations. In each of these 5,000 permutations we randomly changed the sign of the voxel value of each subject and then performing one-sample *t*-test. Note that, a whole-brain mask was used for obtaining the group-level activation map by painful sensation (i.e., Contrast 1) and the group-level activation map by tactile sensation (i.e., Contrast 2). Once the group-level painful and tactile activation maps were obtained, a union mask was created by taking the union of the thresholded painful and tactile activation maps and then used as a mask for determining the corrected *P* values of each voxel of the group-level difference map (i.e., Contrast 3). In addition, we also generated a conjunction map based on the thresholded group-level painful activation map and tactile activation map by taking the overlap of the two thresholded maps.

To further visualize the differences in fMRI responses to painful and tactile stimuli in the brain areas detected by the above Contrast 3, we extracted the time courses of raw fMRI signals (after preprocessing) of each identified cluster of each trial and then averaged across trials and participants for painful and tactile conditions separately. Although the interval between the stimulus and the rating period was randomized (between 3 s and 13 s) within a trial, in some brain areas the stimulus-evoked fMRI responses may temporally overlap with the fMRI responses elicited by the rating process (e.g., button press to indicate the rating on the VAS). To remove these overlapping responses, we calculated an average time course of fMRI signals of the rating period for each identified brain area and then removed it from the time course of the fMRI signals of each condition for the same brain area. Only intensity-matched painful and tactile stimuli were used in this time course analysis.

#### 2.5.3 Analysis (3): Model-free assessment of the time courses of BOLD signals during iso-intense painful and tactile stimulation

Although the voxel-wise GLM analysis offers a good spatial resolution, it faces a severe multiple comparisons problem, and, more importantly, it depends on the assumed haemodynamic response function (HRF), an issue that could bias the results. Therefore, a region-wise model-free analysis was also performed to compare the time courses of fMRI signals between intensity-matched painful and tactile conditions. The whole brain was first divided into regions using pre-defined brain atlases. The same procedure for extracting the time courses of raw fMRI signals described above was then used to obtain the time courses of raw fMRI signals of each condition and each brain region. The area-under-the-curve (AUC) was calculated for the time course of fMRI signals of each condition, each brain region and each participant. The AUCs, as a measure of the fMRI responses to the stimuli, were then statistically compared between painful and tactile conditions using paired *t*-test. The statistical significance was determined using non-parametric permutation testing (n = 5,000) and corrected for multiple comparisons using FWE (*P* < 0.05 corrected). Here, two different brain atlases were used to define brain regions. The first atlas was the combination of the human Brainnetome Atlas (BA) (Fan et al., 2016) (http://atlas.brainnetome.org) and the AAL-cerebellum atlas (i.e., the cerebellar regions in the AAL atlas). The BA divides the cerebrum into 246 regions but does not include the cerebellum. By combining the BA and the AAL-cerebellum atlas, we created a whole-brain atlas (labelled as ‘BA-AAL-cerebellum Atlas’). We noticed that the clusters identified in the GLM analysis were relatively small compared to the regions defined in this atlas. Therefore, a second atlas, which divides the whole brain into 1000 regions by splitting each of the AAL atlas regions into smaller regions (labelled as ‘AAL-1000 Atlas’) (Zalesky et al., 2010) and thus has a much higher spatial resolution (i.e., more and smaller regions are defined in this atlas), was also used. Results obtained from both atlases were reported to provide complementary information as brain areas that are much smaller than the ‘BA-AAL-cerebellum Atlas’ regions could be missed in the first atlas while the second atlas faces more severe multiple comparisons problem. This model-free analysis does not rely on any assumption about the shape, latency and duration of the HRF which has been shown to vary across different brain regions (Lee et al., 1995; Robson et al., 1998) and different types of stimuli (Lui et al., 2008; Mouraux et al., 2011).

#### 2.5.4 Analysis (4): testing the effect of perceived stimulus intensity on the responses of the previously identified ‘modality-preferential’ regions

Analyses 2 and 3 were conducted to identify brain regions showing preferential responses to a given modality (either pain or tactile sensation) while the perceived stimulus intensity was carefully matched between the two modalities. To further test how the perceived stimulus intensity would influence the responses of these brain regions showing a modality preference, we performed a fourth analysis (Analysis 4) to compare the responses of these brain regions when the perceived stimulus intensity of the preferred modality was *lower* than that of the non-preferred modality. That is, for the ‘pain-preferential’ brain regions identified in Analyses 2 and 3, we compared their responses to ‘low-perceived-intensity’ painful stimuli with the responses to ‘high-perceived-intensity’ tactile stimuli. Similarly, for the ‘tactile-processing-preferential’ brain regions, we compared their responses to ‘low-perceived-intensity’ tactile stimuli with the responses to ‘high-perceived-intensity’ painful stimuli. The painful and tactile stimuli were labelled as ‘high perceived intensity’ or ‘low perceived intensity’ for each participant using the follow procedure: all painful and tactile stimuli were first pooled together and then median split into two groups – all stimuli with perceived intensity higher than the median value were labelled as ‘high perceived intensity’ and all stimuli with perceived intensity lower than the median value were labelled as ‘low perceived intensity’. The number of painful stimuli and the number tactile stimuli that were being compared were also equalized by removing some stimuli (near the median value) from the group that had more stimuli. For each of the ‘pain-preferential’ brain regions, the time courses of fMRI responses to ‘low-perceived-intensity’ painful stimuli and the time courses of fMRI responses to ‘high-perceived-intensity’ tactile stimuli were extracted and the corresponding AUCs were calculated, for each participant. Similarly, for each of the ‘tactile-processing-preferential’ brain regions, we also obtained the AUC of the time courses of fMRI responses to ‘low-perceived-intensity’ tactile stimuli and the AUC of the time courses of fMRI responses to ‘high-perceived-intensity’ painful stimuli for each participant. The AUCs of the two conditions (i.e., painful and tactile) were then statistically compared using paired *t*-test. Statistical significance was determined using the same permutation test (n = 5,000) and corrected for multiple comparisons using FWE (*P* < 0.05 corrected) as described in Analysis 3.

## 3 Results

### 3.1 Behavioral data

**Fig. 1:**
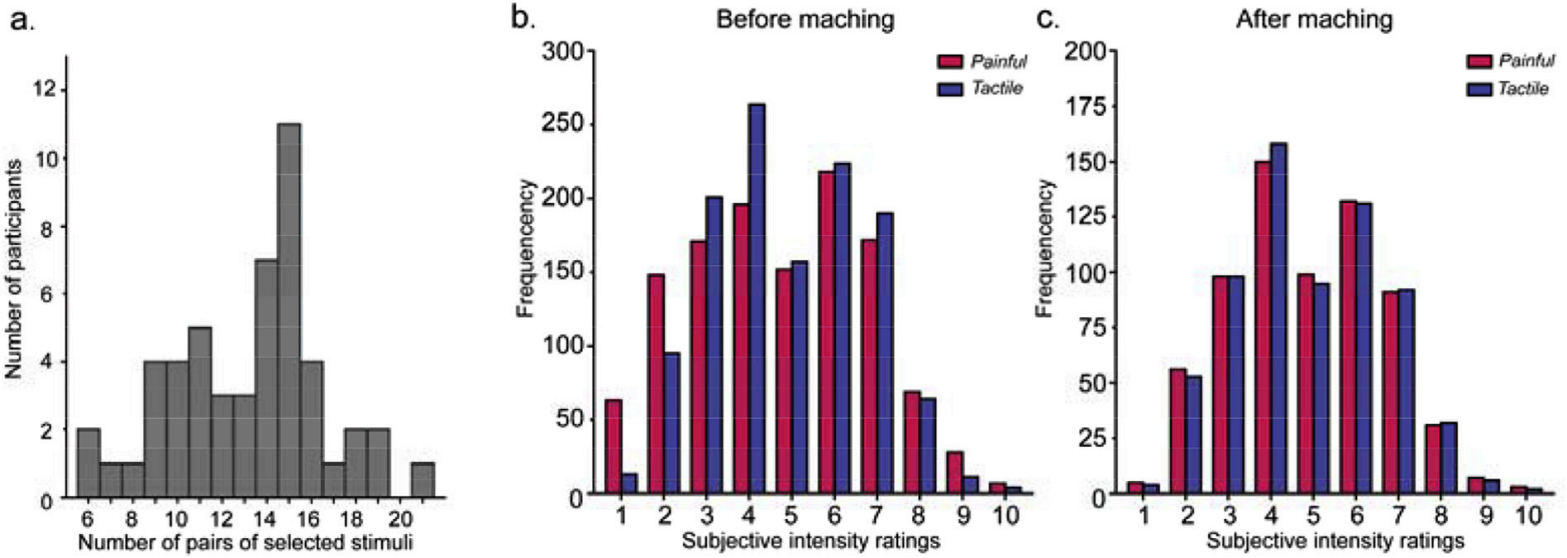
The histogram of the number of selected stimuli with matched perceived intensity (a), the histograms of subjective intensity ratings of *all* painful and tactile stimuli (b) and the histograms of the subjective intensity ratings of the *selected* painful and tactile stimuli with matched perceived intensity (c). In b and c, the histograms for painful stimuli are shown in red and the histograms for tactile stimuli are shown in blue.

The physical and perceived intensities for painful and tactile stimuli at two levels (low vs. high physical intensity) across all participants are summarized in Table 1. To rigorously match the perceived intensity between painful and tactile stimuli, a subset of stimuli was selected in each participant. The number of selected stimuli across participants are summarized in Fig. 1a; the percentage of matched stimuli for every subject is provided in Supplemental Fig. S1. The distribution of subjective intensity ratings of all stimuli and all participants before and after ‘intensity matching’ are displayed in Fig. 1b and c, respectively. The histograms showed that, after the ‘intensity matching’ procedure, perceived intensity was well matched between painful and tactile stimuli.

**Table 1:** The physical and perceived intensities of all painful and tactile stimuli at two levels across participants.

	Painful stimuli		Tactile stimuli	
	Low physical level (Mean ± SD; Range)	High physical level (Mean ± SD; Range)	Low physical level (Mean ± SD; Range)	High physical level (Mean ± SD; Range)
Physical intensity	3.87 ± 0.93J; 1.75–5.75J	4.57 ± 0.93J; 2.25–6.25J	6.27 ± 4.40 mA; 1.00–20.00 mA	13.07 ± 8.11 mA; 2.80–33.00 mA
Perceived intensity	2.76 ± 1.54; 0.00–7.93	5.59 ± 1.51; 2.00–9.90	3.06 ± 1.14; 0.38–7.05	5.65 ± 1.26 1.65–10.00

#### 3.1.1 Analysis (1): brain areas responding to perceived stimulus intensity regardless of stimulus modality

**Fig. 2:**
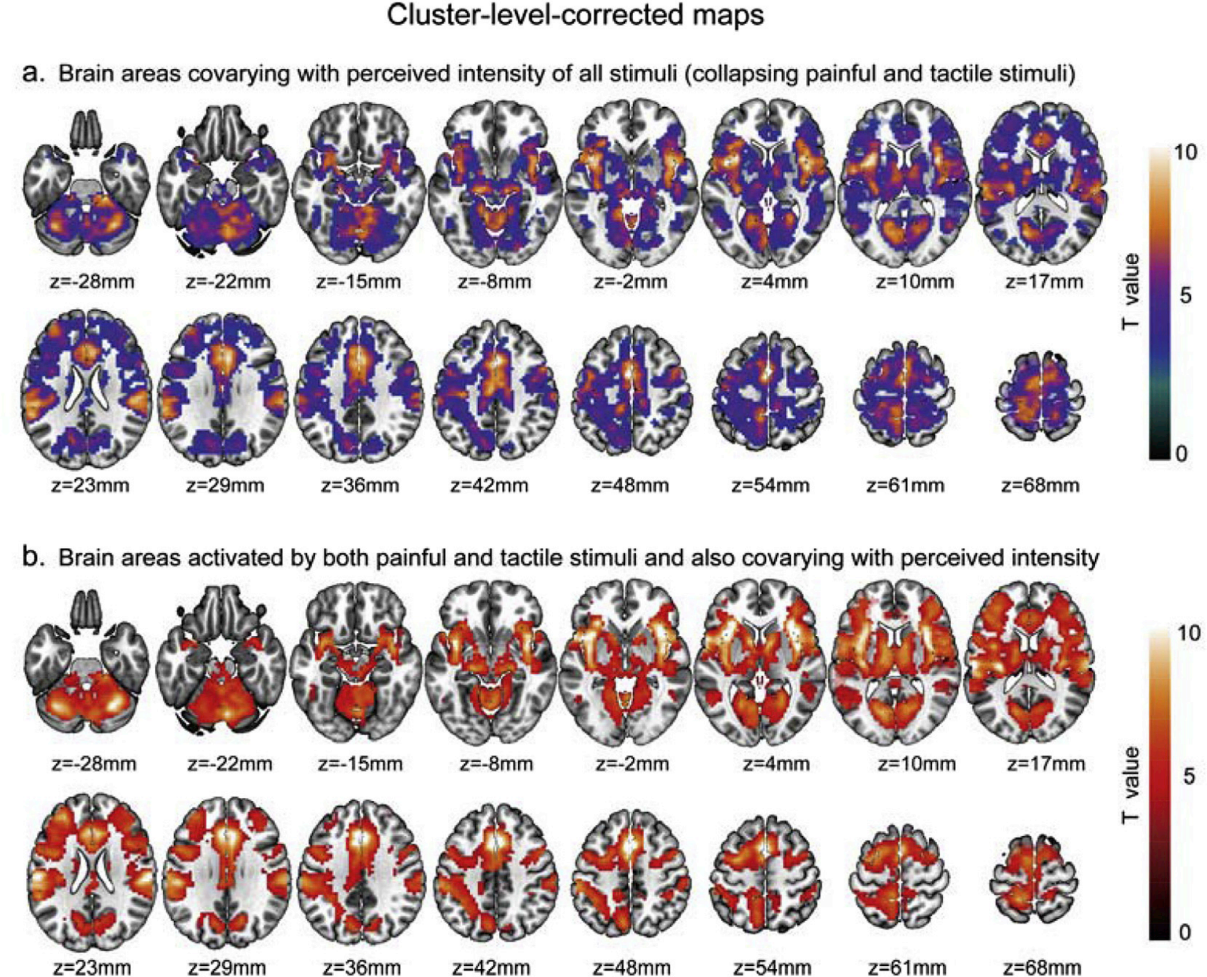
The brain areas in which the neural activity correlated with perceived stimulus intensity regardless of stimulus modality (a) and the conjunct areas activated by both painful and tactile stimuli and at the same time correlated with the perceived stimulus intensity (b). These results were corrected at cluster level.

**Fig. 3:**
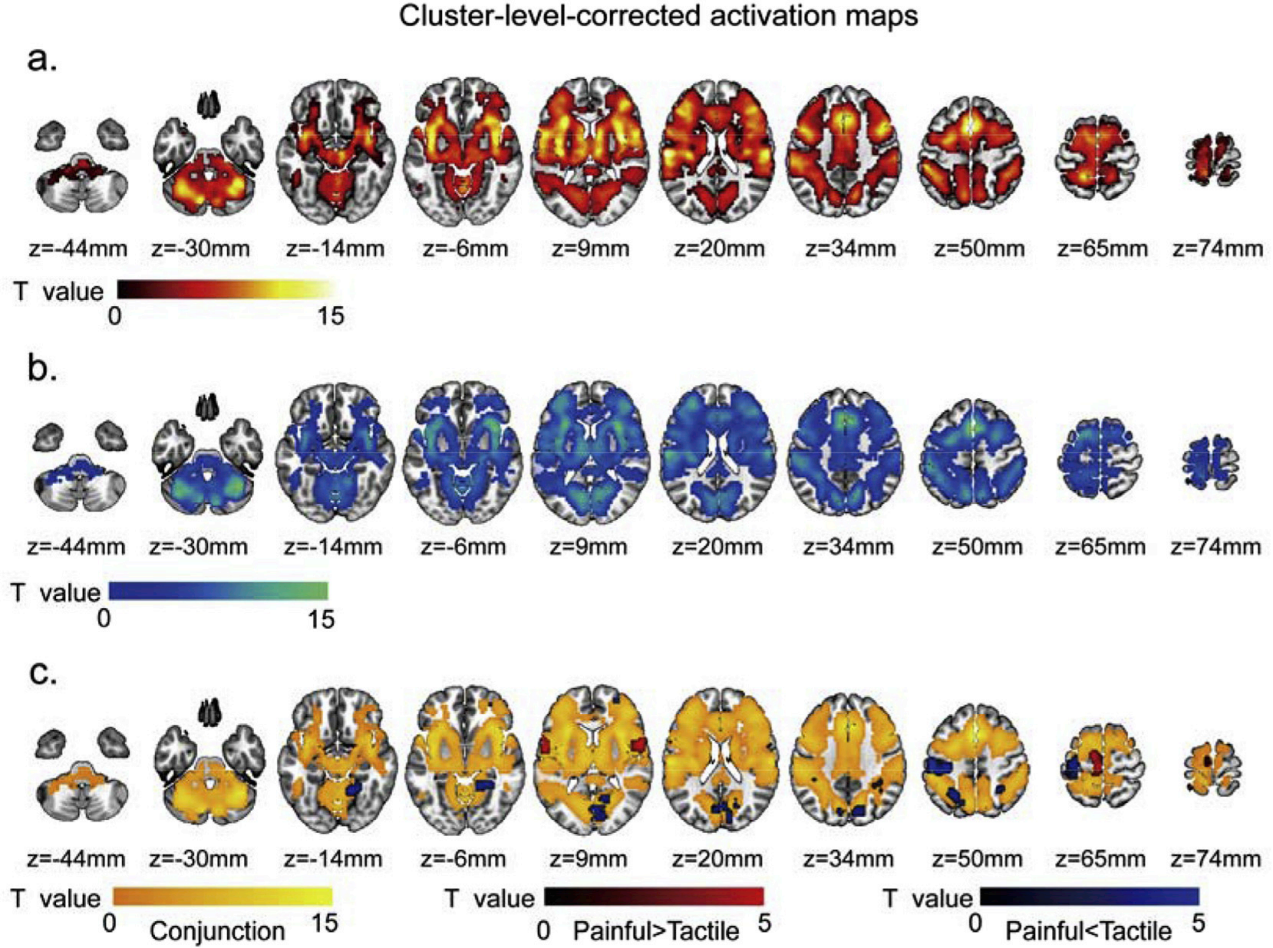
Results of GLM analyses obtained using non-parametric permutation test and corrected using FWE at cluster level (*P* < 0.05 corrected; cluster defining threshold *P* < 0.001): (a) activation map by ‘intensity-matched’ painful sensation, (b) activation map by ‘intensity-matched’ tactile sensation, (c) conjunct activation map (yellow areas) and the areas activated more strongly by painful stimuli than by tactile stimuli (red areas) and the areas activated more strongly by tactile stimuli than by painful stimuli (blue areas).

Using a voxel-wise GLM analysis modelling the perceived stimulus intensity regardless of stimulus modality, we found that the amplitude of fMRI responses correlated with perceived stimulus intensity in a broad network of brain areas. The results, obtained using both cluster-level and voxel-level correction methods are shown in Fig. 2a and Supplemental Fig. S2a, respectively. Within this network, the brain areas with a response most clearly related to perceived stimulus intensity were the primary sensorimotor cortex, the secondary somatosensory cortex, the supplementary motor area, the ACC, the insula, the visual cortex and part of the cerebellum (Fig. 2a). Most of these areas are the core regions often found to be activated by painful stimuli, and often labelled as the “pain matrix” (Garcia-Larrea and Peyron, 2013; Iannetti and Mouraux, 2010, 2015; Tracey and Mantyh, 2007). The distribution of these brain areas is very similar to the activation maps obtained in Analysis 2 in response to painful and tactile stimulation (Fig. 3a and b; see below for detailed results of Analysis 2). The conjunction analysis between these intensity-correlated brain areas (Fig. 2a) and the areas commonly activated by painful and tactile stimuli (Fig. 3c) further confirmed that the neural activity of virtually all brain areas activated by both painful and tactile stimuli also correlated with the perceived stimulus intensity, regardless of stimulus modality (Fig. 2b).

#### 3.1.2 Analysis (2): brain areas commonly and differentially responding to ‘intensity-matched’ painful and tactile stimuli using voxel-wise GLM analysis

**Fig. 4:**
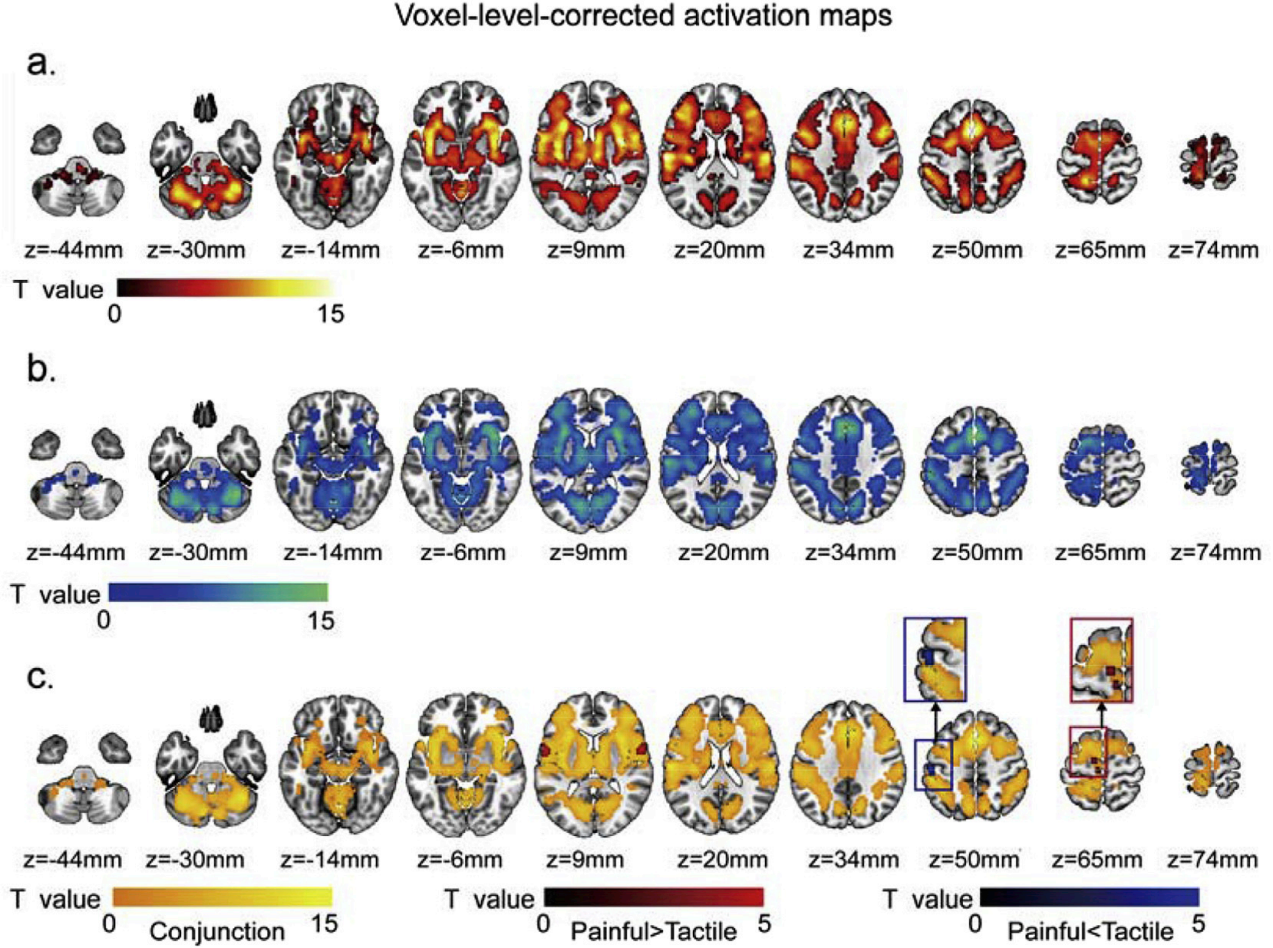
Results of GLM analyses obtained using non-parametric permutation test and corrected using FWE at voxel level (*P* < 0.05 corrected): (a) activation map by ‘intensity-matched’ painful sensation, (b) activation map by ‘intensity-matched’ tactile sensation, (c) conjunct activation map (yellow areas) and the areas activated more strongly by painful than by tactile stimuli (red areas) and the areas activated more strongly by tactile than by painful stimuli (blue areas).

We performed a second GLM analysis to identify the brain areas commonly (by conjunction analysis) and differentially (by contrast analysis) activated by painful and tactile stimuli while the perceived stimulus intensities were matched. The results of the different contrast and conjunction analyses obtained using non-parametric permutation testing are shown in Fig. 3a–c (corrected at *P* < 0.05 cluster-level) and in Fig. 4a–c (corrected at *P* < 0.05 voxel-level): (1) activation by intensity-matched painful stimuli (Fig. 2, Fig. 3, Fig. 4) activation by intensity-matched tactile stimuli (Fig. 3, Fig. 4) conjunction of the activation by both intensity-matched painful and tactile stimuli (yellow areas in Fig. 3, Fig. 4) differences in activation between intensity-matched painful and tactile stimuli (red and blue areas in Fig. 3, Fig. 4c).

The activation maps of the responses elicited by transient painful and tactile stimuli (Fig. 3, Fig. 4a and b), as well as their conjunct map (yellow areas in Fig. 3, Fig. 4c), confirmed that both stimuli elicit responses in a largely similar and widely distributed network of brain areas, similar to what we reported in our first study about the specificity of responses of the “pain matrix” (Mouraux et al., 2011). Importantly, differences in intensity of activation between painful and tactile stimulation were detected, and in both directions (Fig. 3, Fig. 4c). Three clusters located in the bilateral Rolandic operculum and the left supplemental motor area (SMA) showed stronger activation during painful stimulation than during tactile stimulation (red areas in Fig. 3, Fig. 4c), and one cluster located in the left postcentral gyrus showed stronger activation during tactile stimulation than during painful stimulation (blue areas in Fig. 3, Fig. 4c). Both clusters were identified after using the voxel-level and cluster-level corrections (Table 2). Cluster-level correction detected five additional clusters with stronger activation during tactile stimulation than during painful stimulation; these were located in the calcarine cortex, the right cerebellum, the right parietal inferior gyrus, the left parietal superior gyrus and the right frontal middle gyrus (blue areas in Fig. 3c, Table 2). Similar results were also obtained using other correction methods based on conventional RFT at cluster-level or voxel-level, as shown in Supplemental Figs. S3–S4.

**Table 2:** Clusters showing significantly different responses to intensity-matched painful and tactile stimuli identified by non-parametric permutation test and corrected at cluster level. L: left; R: right.

Regions	Cluster size (voxels)	Peak intensity (T/*P* value)	Coordinates (x, y,z)
*Painful > Tactile*			
Rolandic Operculum (R)	84	6.838/5.379E-9	60, 6, 9
Rolandic Operculum (L)	70	7.389/7.401E-10	−57, 3, 9
Supplemental Motor Area (L)	100	5.403/9.116E-7	−9, −9, 69
*Painful < Tactile*			
Postcentral Gyrus (L)	320	−5.544/5.544E-7	−54, −27, 54
Calcarine (L, R)	495	−4.769/8.210E-6	24, −51, −15
Cerebellum (R)	53	−5.499/6.499E-7	21, −51, −18
Parietal Inferior Gyrus (R)	72	−4.267/4.405E-5	30, −48, 39
Parietal Superior Gyrus (L)	100	−4.470/2.251E-6	−27, −57, 54
Frontal Middle Gyrus (R)	55	−4.820/6.899E-6	30, 54, 0

**Fig. 5:**
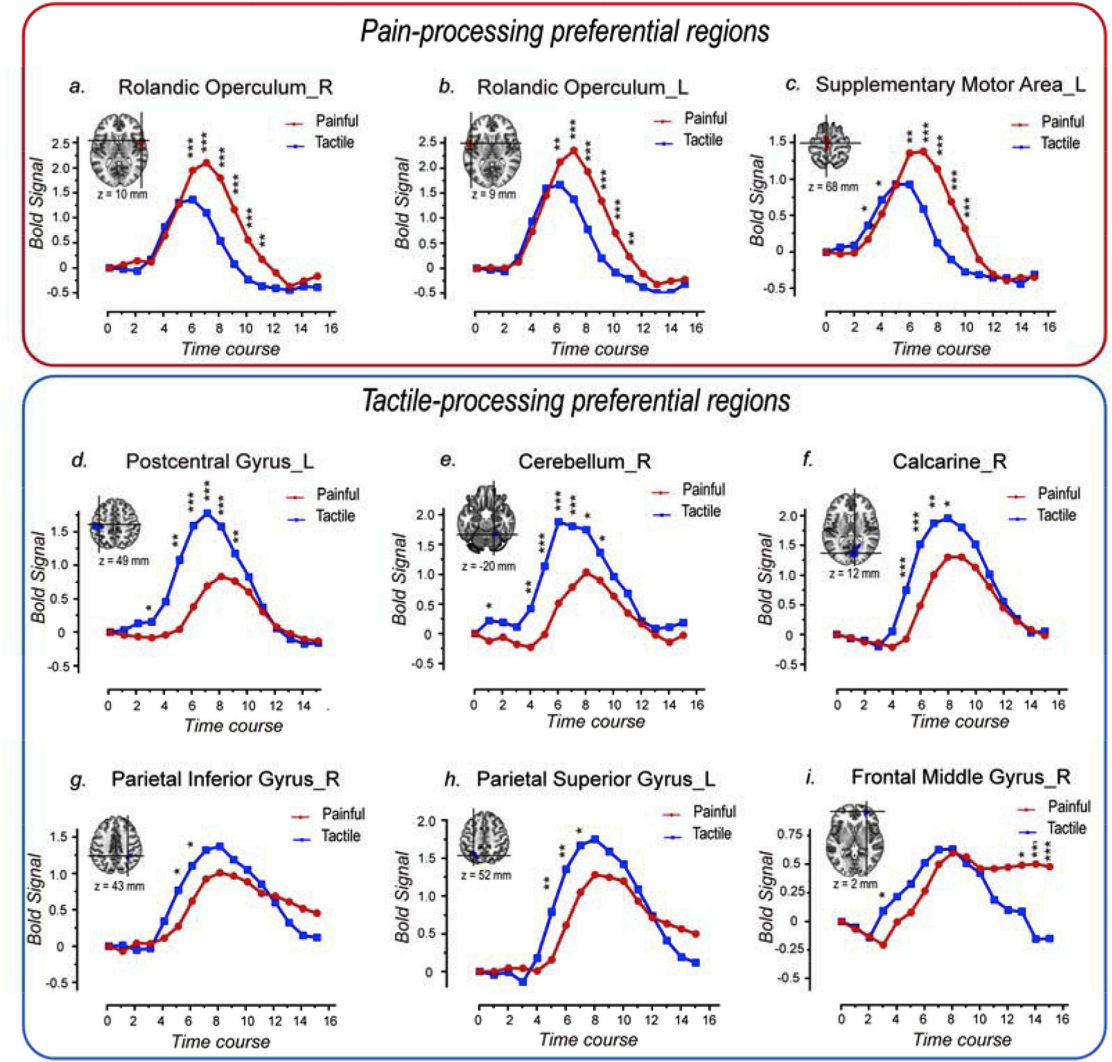
Time courses of the fMRI signals extracted from the nine clusters activated differently by painful and tactile stimuli detected using voxel-wise GLM analysis (red: painful; blue: tactile). Three clusters were identified as ‘painful>tactile’ (a-c): they were located in the right Rolandic operculum (a), the left Rolandic operculum (b), and the left supplemental motor area (c) and showed greater signal amplitude and longer duration for painful sensation than for tactile sensation. Six clusters were identified as ‘tactile>painful’ and located in the left postcentral gyrus (d), the right cerebellum (e), the right calcarine (f), the right parietal inferior gyrus (g), the left parietal superior gyrus (h) and the right frontal middle gyrus (i). The first five clusters showed greater signal amplitude for tactile than for painful sensation (d-h). For the sixth cluster located in the right frontal middle gyrus (i), although detected as ‘tactile>painful’ by GLM, the fMRI signals increased to a similar amplitude after both painful and tactile stimuli but did not return to baseline for painful stimulation. Paired *t*-test was also performed to compare the signal amplitude between painful and tactile conditions for each time point, and the time points at which the fMRI signal amplitudes were significantly different are indicated by asterisks. *, P < 0.05; **, P < 0.01; ***, P < 0.001.

We further extracted the time courses of the fMRI signals of the nine clusters (Fig. 5a–i) detected by the non-parametric permutation test combined with cluster-level correction (Fig. 3c), to examine how differently they responded to painful and tactile stimuli. The results showed that all clusters responded to both painful and tactile stimuli but the response amplitude and/or duration, were different in the two conditions. In general, the fMRI response elicited by painful stimuli had larger amplitude and lasted longer in the three clusters detected to respond more strongly to pain (Fig. 5a–c). The fMRI response elicited by tactile stimuli had larger amplitude in all clusters detected to respond more strongly to tactile stimuli (Fig. 5d–h), with the exception of the cluster in the frontal middle gyrus, in which the fMRI responses elicited by painful and tactile stimuli had similar amplitude but lasted longer in response to painful stimuli (Fig. 5i). The fMRI responses at each time point (Fig. 5) and the AUC of the time courses (Fig. 7a) were also compared between the painful and tactile conditions for each cluster using paired *t*-test.

Notably, the peak of the responses to painful stimuli occurred later than that of the responses to tactile stimuli by one or two time points in five clusters located in the bilateral Rolandic operculum, the left SMA, the left postcentral gyrus and the right cerebellum (Fig. 5a–e). This difference in peak time between responses to painful and tactile stimuli is likely to be due to the difference in conduction time of peripheral and central nervous system between nociceptive and tactile information (Iannetti et al., 2003; Inui et al., 2003; Mouraux et al., 2010; Ploner et al., 2006). Also, the peak of the responses to both painful and tactile stimuli occurred later in most of the ‘tactile-processing-preferential’ areas (Fig. 5f–i) than the ‘pain-processing-preferential’ areas (Fig. 5a–c).

#### 3.1.3 Analysis (3): brain areas differentially activated by ‘intensity-matched’ painful and tactile stimuli using region-wise model-free analysis

**Fig. 6:**
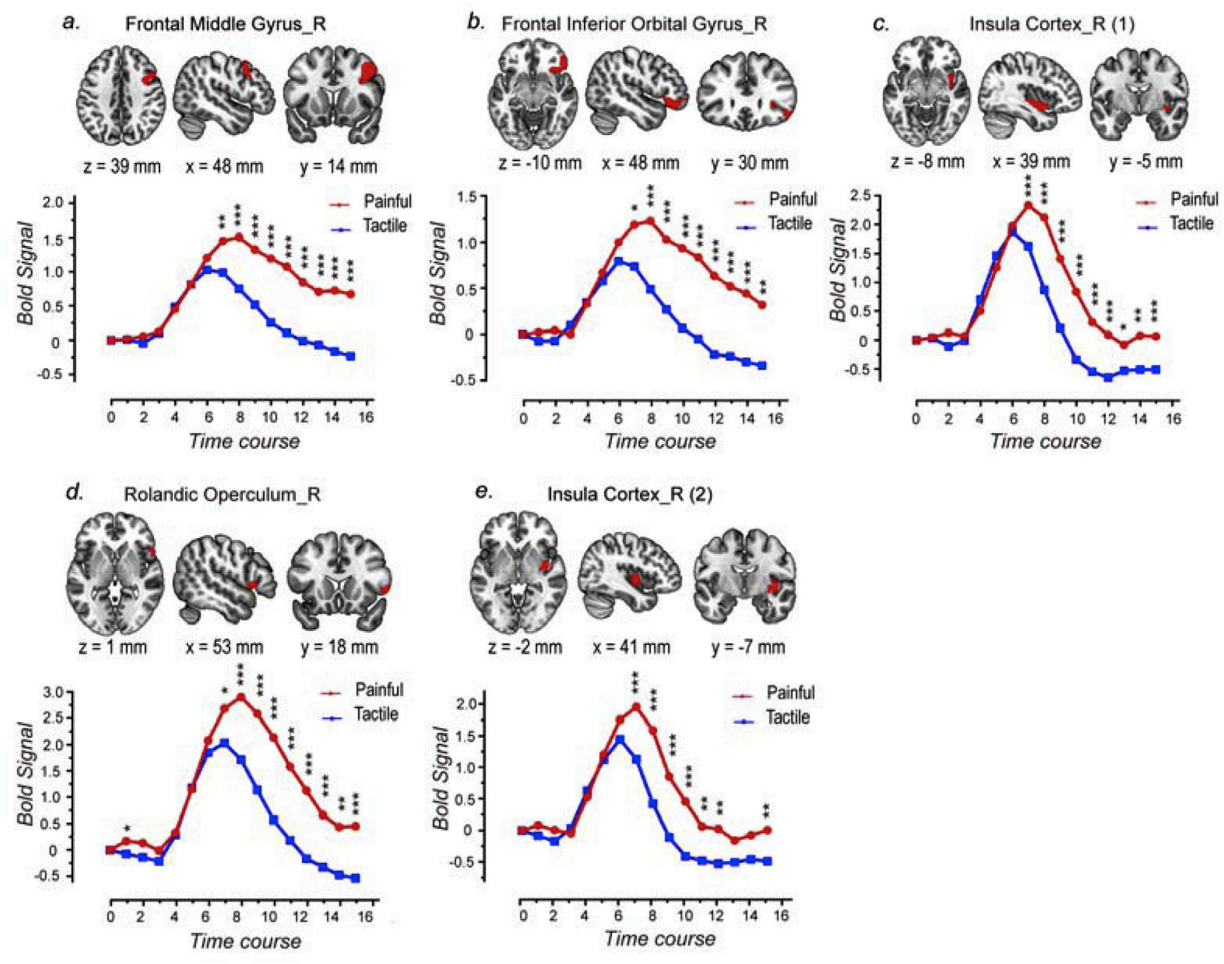
The locations of the brain regions responding more strongly to painful than to tactile stimuli, along with their time courses of the fMRI responses under the two conditions, identified by the region-wise model-free analysis using the ‘BA-AAL-cerebellum’ atlas (a-c) and using the ‘AAL-1000’ atlas (d-e). Paired *t*-test was also performed to compare the signal amplitude between painful and tactile conditions for each time point, and the time points at which the fMRI signal amplitudes were significantly different are indicated by asterisks. *, *P* < 0.05; **, *P* < 0.01, ***, *P* < 0.001.

All regions detected by the model-free analysis showed stronger responses (i.e., higher amplitude) to painful stimuli than to tactile stimuli (Fig. 6). Using the ‘BA-AAL-cerebellum’ atlas, three regions responded more strongly to painful than to tactile stimuli: the right frontal middle gyrus, the right frontal inferior orbital gyrus and the right insula (see Fig. 6a–c for their exact spatial locations). Using the ‘AAL-1000’ atlas, two regions were detected: the right Rolandic operculum and the right insula (see Fig. 6d and e for their exact spatial locations). The time courses of the fMRI signals in these regions are shown in Fig. 6. Similarly to what was observed in the GLM analysis, fMRI responses to painful stimuli had larger amplitude, longer duration and peaked later than fMRI responses to tactile stimuli (Fig. 6). No regions were detected to respond more strongly to tactile stimuli than to painful stimuli.

#### 3.1.4 Analysis (4): the responses of regions with a modality preference were affected by perceived stimulus intensity

Eight clusters were identified as ‘pain-preferential’ areas in Analyses 2 and 3. The time courses of the responses elicited in these clusters by ‘low-perceived-intensity’ painful stimuli and by ‘high-perceived-intensity’ tactile stimuli are shown in Fig. 8, and the results of statistical comparisons were shown in Fig. 10a. While only one cluster in the right insula showed a stronger response to tactile than to painful stimuli (p = 0.024; Fig. 10a), most (seven out of eight) clusters showed a trend of higher responses to ‘high-intensity’ tactile stimuli than to ‘low-intensity’ painful stimuli. These results indicate that the responses of these areas were mainly determined by stimulus intensity, and that their preference to pain can only be observed when stimulus intensity is carefully matched. The only exception was the cluster located in the frontal middle gyrus which still showed higher and longer-lasting responses to painful stimuli than to tactile stimuli even when painful stimuli were perceived less intense than tactile stimuli, although the difference in AUC did not reach the significance level.

**Fig. 7:**
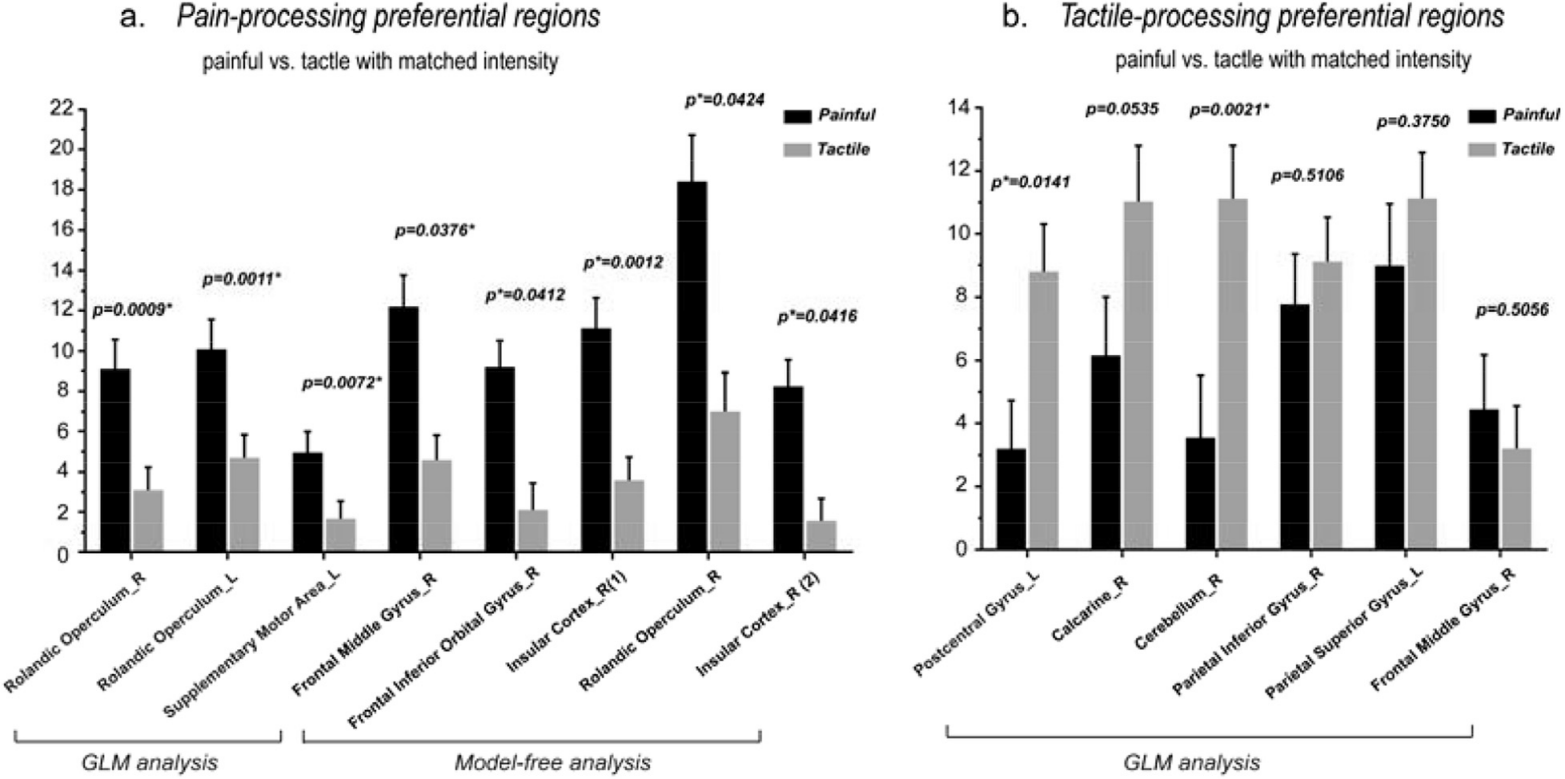
Comparisons of the area under curve (AUC) of the time courses of the fMRI signals between intensity-matched ‘painful’ condition and ‘tactile’ condition for the eight clusters that were identified as ‘painful > tactile’ (a) and for the six clusters that were identified as ‘tactile > painful’ (b). The AUCs were compared between painful and tactile conditions using paired *t*-test. The error bars indicate the standard error of mean.

**Fig. 8:**
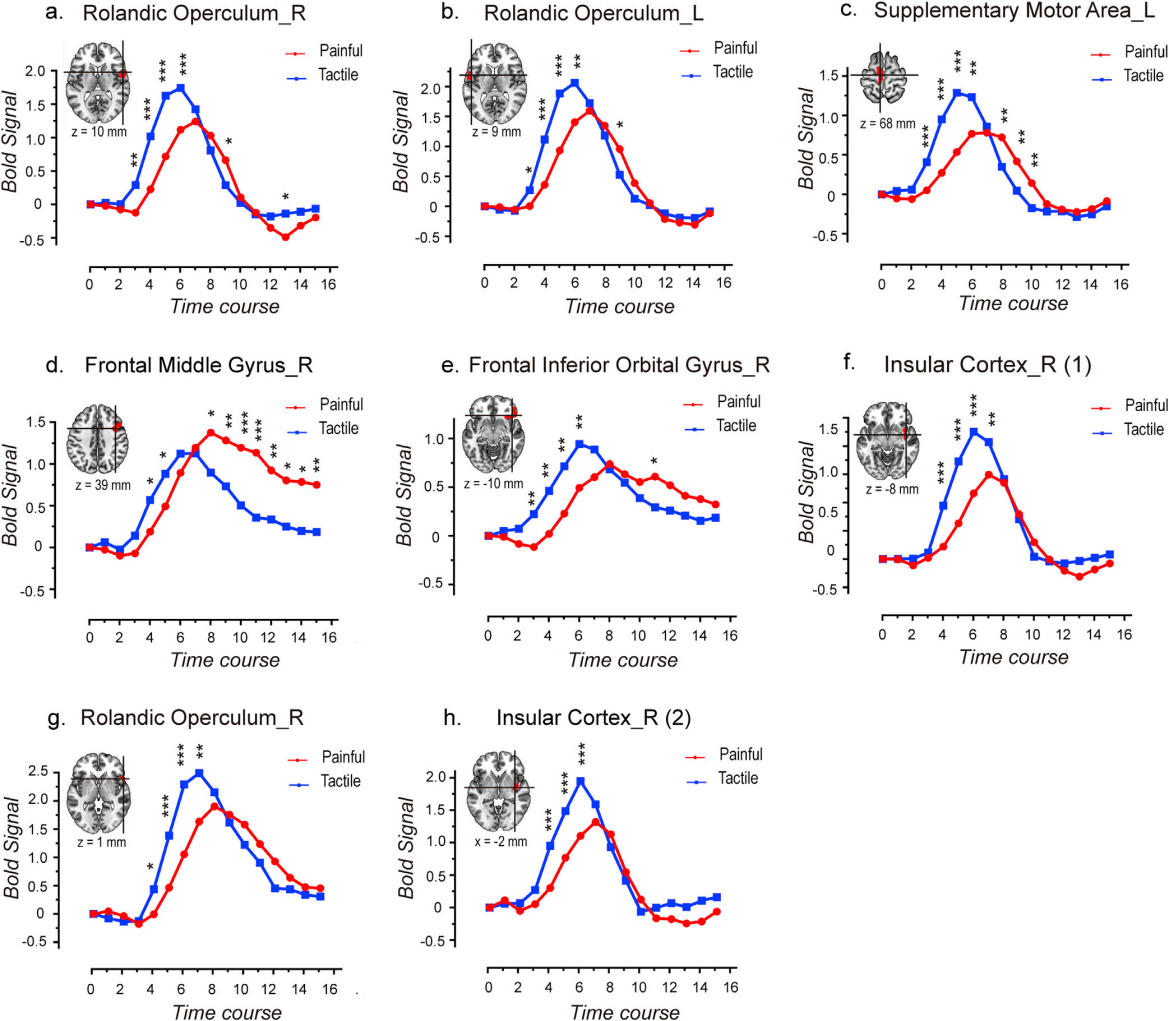
Comparisons of the fMRI time courses between ‘low-perceived-intensity’ painful stimuli (in red) and ‘high-perceived-intensity’ tactile stimuli (in blue) for the eight ‘pain-preferential’ clusters identified in Analyses 2 and 3. Paired *t*-test was performed to compare the signal amplitude between painful and tactile conditions for each time point, and the significance are indicated by asterisks (*, P < 0.05; **, P < 0.01; ***, P < 0.001).

**Fig. 9:**
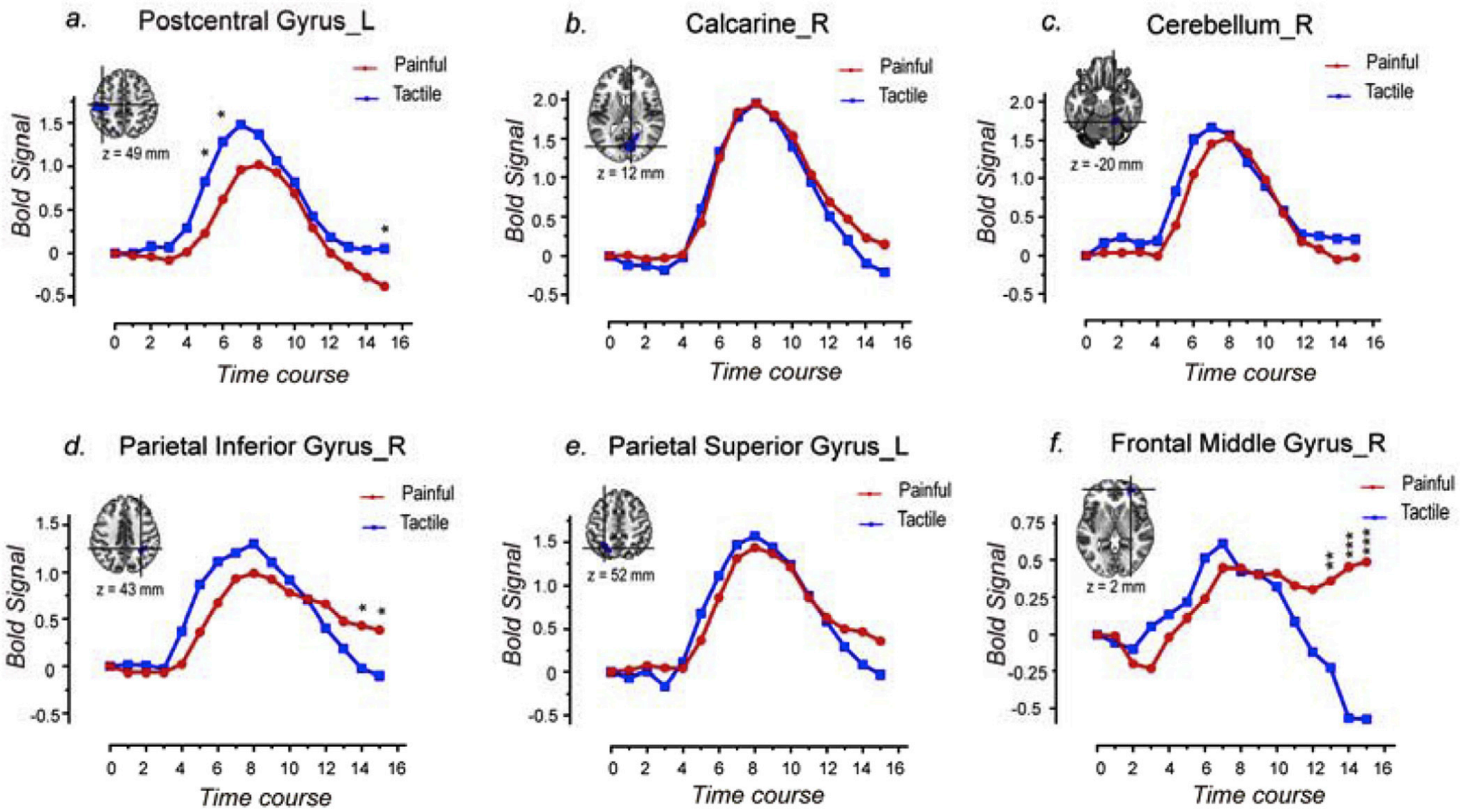
Comparisons of the fMRI time courses between ‘low-perceived-intensity’ tactile stimuli (in blue) and ‘high-perceived-intensity’ painful stimuli (in red) for the six ‘tactile-processing-preferential’ clusters identified in Analyses 2 and 3. Paired *t*-test was performed to compare the signal amplitude between painful and tactile conditions for each time point, and the significance are indicated by asterisks (*, P < 0.05; **, P < 0.01; ***, P < 0.001).

**Fig. 10:**
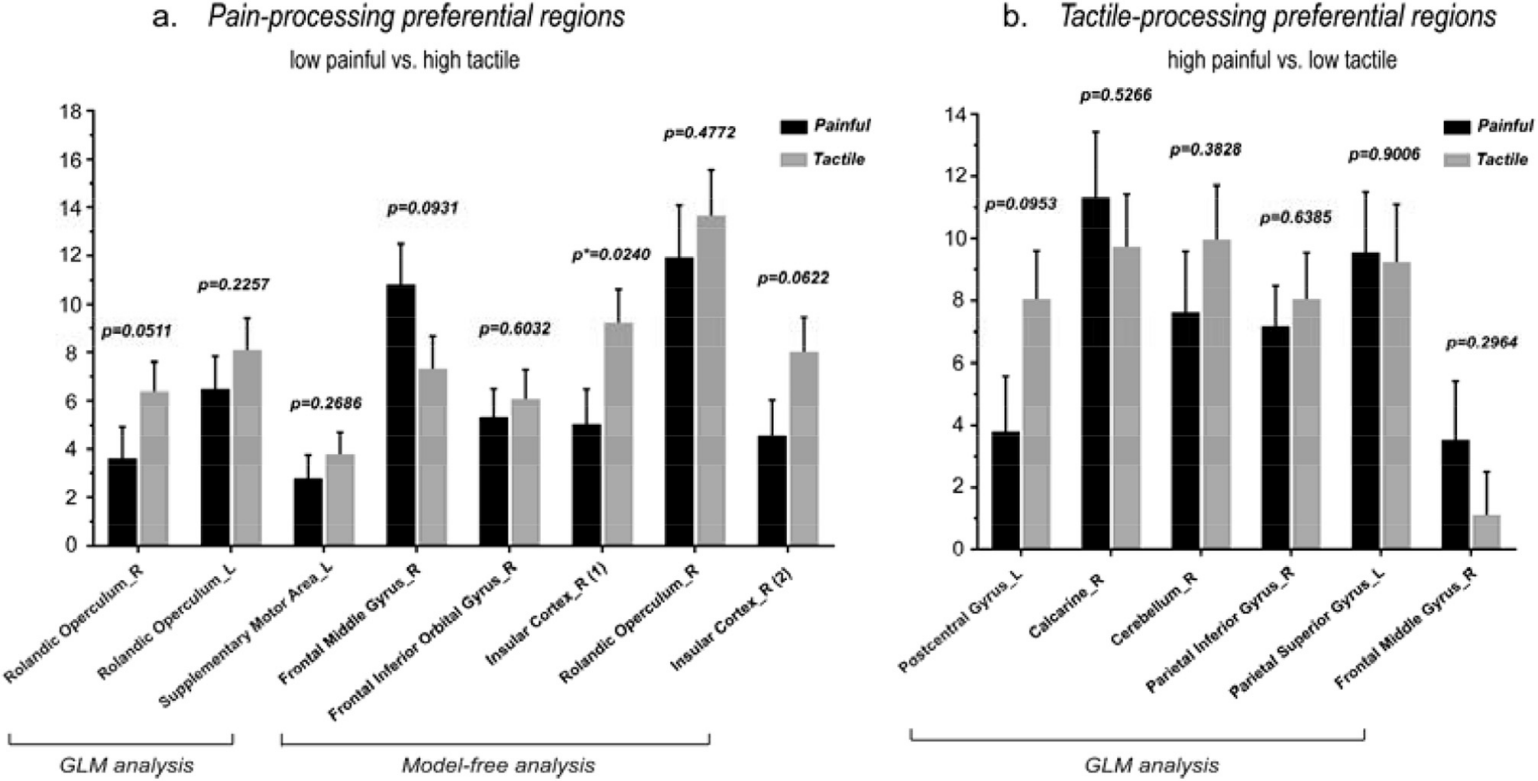
Comparisons of the AUC of the time courses of the fMRI signals between ‘low-perceived-intensity painful’ condition and ‘high-perceived-intensity tactile’ condition for the eight clusters that were identified as ‘painful > tactile’ (a) and comparisons of the AUC of the time courses of the fMRI signals between ‘low-perceived-intensity tactile’ condition and ‘high-perceived-intensity painful’ condition for the six clusters that were identified as ‘tactile > painful’ (b). The AUCs were compared between painful and tactile conditions using paired *t*-test. The error bars indicate the standard error of mean.

Six clusters were identified as ‘tactile-processing-preferential’ areas in Analyses 2 and 3. The time courses of the responses elicited in these clusters by ‘low-perceived-intensity’ tactile stimuli and by ‘high-perceived-intensity’ painful stimuli are shown in Fig. 9, and the results of statistical comparisons are shown in Fig. 10b. We observed that none of these clusters had significantly different AUCs between painful and tactile conditions. Indeed, five out of six areas had similar time courses of fMRI responses to ‘low-intensity’ tactile stimuli and to ‘high-intensity’ painful stimuli, indicating that the response preference of these areas was canceled out by the difference in perceived stimulus intensity. The only exception was the cluster located in the postcentral gyrus which still showed a trend of higher responses to tactile stimuli than to painful stimuli even when tactile stimuli were perceived less intense than painful stimuli.

## 4 Discussion

In this study we characterized the differences in the brain responses elicited by transient painful and tactile stimuli. Given that the difference in perceived stimulus intensity is a major confound when comparing the responses elicited by such stimuli (Coghill et al., 1999; Iannetti et al., 2008), we ensured that perceived stimulus intensity was strictly matched between the two modalities on a trial-by-trial basis. FMRI data were explored using both voxel-wise GLM analysis and region-wise model-free analysis, and the robustness of the GLM results were also tested using different multiple comparisons correction methods. We found four main results. First, brain areas activated by transient painful stimuli were also activated by transient tactile stimuli (Fig. 3, Fig. 4c, yellow), confirming a number of previous findings (Apkarian et al., 2005; Mouraux et al., 2011; Stern et al., 2006; Tracey and Mantyh, 2007; Wager et al., 2013). Second, the amplitude of neural activity in all these areas correlated strongly with perceived stimulus intensity (Fig. 2b), highlighting the importance of matching perceived intensity when comparing brain responses to painful and tactile stimuli. Third, when perceived intensity was rigorously matched, several brain areas responded differentially to painful and tactile stimuli: some responded more strongly to painful stimuli (Fig. 3, Fig. 4c, red) and some responded more strongly to tactile stimuli (Fig. 3, Fig. 4c, blue). Fourth, the responses of these ‘modality-preferential’ brain areas were determined by both stimulus modality and stimulus intensity. These results indicate that, although sudden painful and tactile stimuli activate the same set of brain areas and the perceived stimulus intensity is the most important determinant factor of their responses, different areas may have different preference in processing painful vs. tactile sensations.

### 4.1 Identification of pain-preferential neural activities requires rigorous matching of stimulus intensity

To identify brain areas that respond preferentially to pain, it is mandatory to compare brain responses to painful and non-painful stimuli. Here, we chose non-painful tactile stimuli as control, because they belong to the somatosensory domain but do not elicit painful sensations and thus provide a strict control. However, a key confound in such analyses is the perceived stimulus intensity, which has been often neglected in previous studies (for example, when the brain responses elicited by high-temperature painful heat were contrasted with those elicited by low-temperature warmth (Coghill et al., 1999; Wager et al., 2013)). Our results clearly demonstrate that in virtually all brain areas the responses evoked by painful and tactile stimuli depend on perceived stimulus intensity (Fig. 2b). This result confirms the necessity of matching perceived intensity when performing such comparisons. Here, we aimed to match perceived stimulus intensity rather than physical intensity because it has been shown that there is a large inter-subject variability of sensory sensitivity (Coghill et al., 2003; Hu and Iannetti, 2019; Nielsen et al., 2009). In other words, two stimuli with identical physical intensity could are typically perceived very differently by different participants. Note that the largest part of the neural activity elicited by transient nociceptive stimuli is in fact determined by stimulus saliency (Mouraux and Iannetti, 2018). Although ‘perceived stimulus intensity’ and ‘stimulus saliency’ are two different concepts and can be psychophysically distinguished from each other in several contexts (Iannetti et al., 2008; Ronga et al., 2013), the two measures are highly correlated and indistinguishable in most scenarios, including the present experimental design. It should also be noted that, to ensure a rigorous match of stimulus intensity between painful and tactile conditions, we had to discard some trials in each participant, which resulted in unequal number of trials across participants, a factor that might have affected the statistical significance of the results.

### 4.2 Transient painful and tactile stimuli largely activate the same set of brain areas

Our observation that the brain areas activated by painful stimuli can also be activated by tactile stimuli (yellow areas in Fig. 3, Fig. 4c) confirms our previous finding from a different dataset (Mouraux et al., 2011). Importantly, all clusters identified to respond differentially to painful and tactile stimuli (red and blue areas in Fig. 3, Fig. 4c) were located inside the conjunct activated areas. This indicates that, although these brain areas were detected to respond differentially to intensity-matched painful and tactile stimuli (see the results of Analyses 2 and 3), they did not exclusively respond to either modality, but responded to both modalities. This suggests that pain-specific information is not encoded in any exclusively dedicated brain region. The fact that only transient stimuli were used makes the current results cannot be generalized to longer-lasting painful stimuli.

### 4.3 Certain brain areas respond more strongly to painful stimuli

Certain brain areas responded differentially to painful and tactile stimuli even when perceived stimulus intensity was strictly matched between the two conditions. Among these areas, the bilateral parietal operculum, the left SMA, the right insula and the right prefrontal areas were found to respond more strongly to painful stimuli than to tactile stimuli (Table 2, Fig. 5, Fig. 6).

The involvement of the parietal operculum (largely corresponding to the secondary somatosensory area, S2) and the insula in somatosensory processing is well known and reported in a large number of studies (Corradi-Dell'Acqua et al., 2016; Frot et al., 2007; Frot and Mauguiere, 2003; Iannetti et al., 2005a; Isnard et al., 2011; Peyron et al., 2002). The particular involvement of the operculoinsular areas in human pain processing has been suggested in a previous study utilizing a variety of neuroimaging techniques including PET, fMRI, ERP (event-related potentials) from scalp EEG and intracerebral recordings of evoked potentials (Peyron et al., 2002). It has also been shown that electrical stimulation of the operculoinsular cortex can elicit painful sensations (Afif et al., 2010; Isnard et al., 2011; Ostrowsky et al., 2002). However, adequate control stimuli with matched intensity were lacking in these previous studies. The current results obtained from intensity-matched stimuli provided more solid evidence supporting that the operculoinsular cortex respond preferentially to nociceptive stimuli.

The SMA contralateral to the stimulated site also responded more strongly to painful than to tactile stimuli. The SMA is traditionally associated with motor-related functions, especially more complex movements, such as motor sequence planning and motor learning (Nachev et al., 2008). Stimulation of the SMA could evoke movements or even just the urge to move or movement inhibition (Freund, 1996; Fried et al., 1991). Therefore, the observed stronger activation in the left SMA (i.e., contralateral to the stimulated side) during painful stimulation on the right foot may be related to an intrinsically closer relationship between pain and the need to execute defensive motor response (Moayedi et al., 2015; Novembre et al., 2018): although there is no explicit movement directly related to painful or tactile stimuli in the present experiment, painful stimuli could implicitly elicit, consciously or subconsciously, an urge for an escape action to a greater extent than tactile stimuli, even though the perceived stimulus intensity is strictly matched between the two conditions.

The model-free regional analysis further identified two lateral prefrontal areas (the frontal middle gyrus and the frontal inferior orbital gyrus) responding more and for a longer time to painful than to tactile stimuli. Even when painful stimuli were less intense than tactile stimuli, the frontal middle gyrus still showed a trend of stronger and longer response to painful than to tactile stimuli (Fig. 8, Fig. 10a). These lateral prefrontal areas are related to high-level cognitive functions, such as working memory (Levy and Goldman-Rakic, 2000; Owen, 1997; Owen et al., 1996, 1998), episodic memory (Desgranges et al., 1998; Speck et al., 2000), attention (Corbetta et al., 2008; Corbetta and Shulman, 2002; Fox et al., 2006) and emotional processing (Banks et al., 2007; Olson et al., 2007; Zahn et al., 2009). As pain is multidimensional, including not only sensory components but also affective and cognitive components, the initial sensory components of pain sensations could further elicit a series of higher cognitive activities which might underlies the higher and longer responses we observed in these lateral prefrontal areas. Though it is also worth noting that the painful sensation elicited by laser stimuli might last longer than the tactile sensation elicited by electrical stimuli, the difference in the duration of fMRI responses was not observed ubiquitously in the activated brain areas, suggesting that our results cannot be explained by the difference in the duration of sensory input alone.

Interestingly, GLM analysis showed that the cluster located in the right frontal middle gyrus responded more strongly to tactile than to painful stimuli (Fig. 5i). However, this cluster had also longer responses to painful than to tactile stimuli (Fig. 5i). The reason that GLM detected this area to respond more strongly to tactile stimuli is, at least partly, due to the fact that the GLM analysis relies on the assumption of the shape of the HRF: the waveform of BOLD signals elicited by tactile stimuli followed a regular increasing-decreasing changes (i.e., bell shape) and thus fit better with the assumed shape of the HRF; whereas the waveform of BOLD signals elicited by painful stimuli remained at high level after reaching the peak.

### 4.4 Other brain areas respond more strongly to tactile stimuli

Five clusters located in the left postcentral gyrus, the calcarine cortex, the right cerebellum, the right parietal inferior gyrus and the left parietal superior gyrus responded more strongly to tactile than to painful stimuli. This was observed both using voxel-wise GLM analysis and model-free analysis of BOLD time courses (Fig. 5d–h). Furthermore, these areas did not show stronger responses to painful stimuli even when painful stimuli were perceived as more intense than tactile stimuli (Fig. 9), especially for the left postcentral gyrus which still showed a trend of stronger responses to tactile stimuli with low perceived intensity (Fig. 9, Fig. 10b). Another interesting finding is that the peak of the responses to both painful and tactile stimuli occurred later in the ‘tactile-processing-preferential’ areas (e.g., at the 8th TR after stimulus onset for painful and tactile stimulation; Fig. 5f–h) than in the ‘pain-processing-preferential’ areas (i.e., at the 7th and 6th TR after stimulus onset for painful and tactile stimulation, respectively; Fig. 5a–c). This peak time difference observed between the two different groups of brain areas also suggests that these brain areas serve different functions in processing painful and tactile information. These observations are somewhat unexpected and requires further investigation.

Note that ‘tactile-processing-preferential’ areas were only detected using voxel-wise GLM analysis but not using other analysis approaches. Many factors can contribute to this discrepancy. For example, different approaches may be sensitive in detecting different types of information: GLM approach does not require a priori definition of brain regions and thus can detect clusters of any shape in the brain, but this approach is based on the assumption of HRF and thus is only sensitive in detecting brain areas where the temporal dynamics of the fMRI responses fits with the presumed HRF (Iannetti and Wise, 2007). On the contrary, the model-free analysis does not require any assumption of the HRF and is thus more sensitive in detecting brain areas with arbitrary temporal dynamics of fMRI responses, whereas it is less powerful in detecting clusters with arbitrary spatial distributions that do not fit the brain parcellation predefined by an atlas.

## 5 Conclusion

By rigorously matching the perceived stimulus intensity and comparing the brain responses to painful and tactile stimuli, we confirm that iso-intense painful and tactile stimulation activate the same set of brain areas. Thus, brain regions exclusively dedicated to encoding pain-specific information are unlikely to exist. Furthermore when perceived intensity is rigorously matched, some of the areas responding to both painful and tactile stimuli show stronger responses to stimuli of either modality, suggesting that different brain areas may preferentially process painful or tactile information. These findings were obtained using very transient heat nociceptive stimuli, and their clinical translation is therefore limited. Further investigations are needed to understand how clinical acute, subacute and chronic pain are specifically represented in the human brain.

## Conflicts of interest

The authors declare no competing financial interests.

## Data and code availability statement

The code supporting the findings of this study are available from the corresponding author upon request.

## Ethics statement

All participants provided written informed consent prior to the experiment, and the experimental procedures were approved by the Medical Research Ethics Committee of Tianjin Medical University.
