# Supplementary material for: Brain regions preferentially responding to transient and iso-intense painful or tactile stimuli
Source: Neuroimage. 2019 May 15;192:52–65. doi: 10.1016/j.neuroimage.2019.01.039 (PMC6503155; doi:10.1016/j.neuroimage.2019.01.039)
Supplement: Multimedia component 1 [file mmc1.docx]

Supplemental Information

for

Brain regions preferentially responding

to transient and iso-intense painful or tactile stimuli

Su Q^1^, Qin W^2^, Yang QQ^1^, Yu CS^2,3^, Qian TY^4^, Mouraux A^5^, Iannetti GD^6^, Liang M^1^*

**Supplemental Figures**


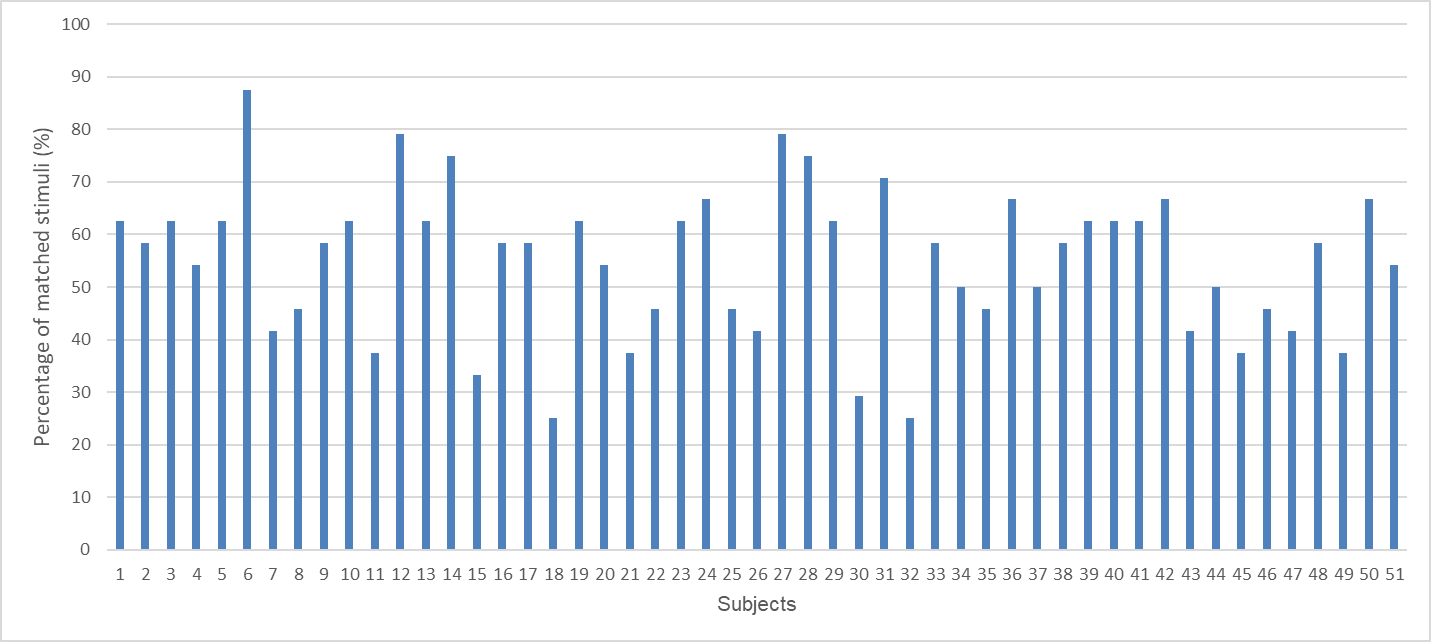


**Supplemental Figure S1.** Percentages of intensity-matched stimuli for every subject.


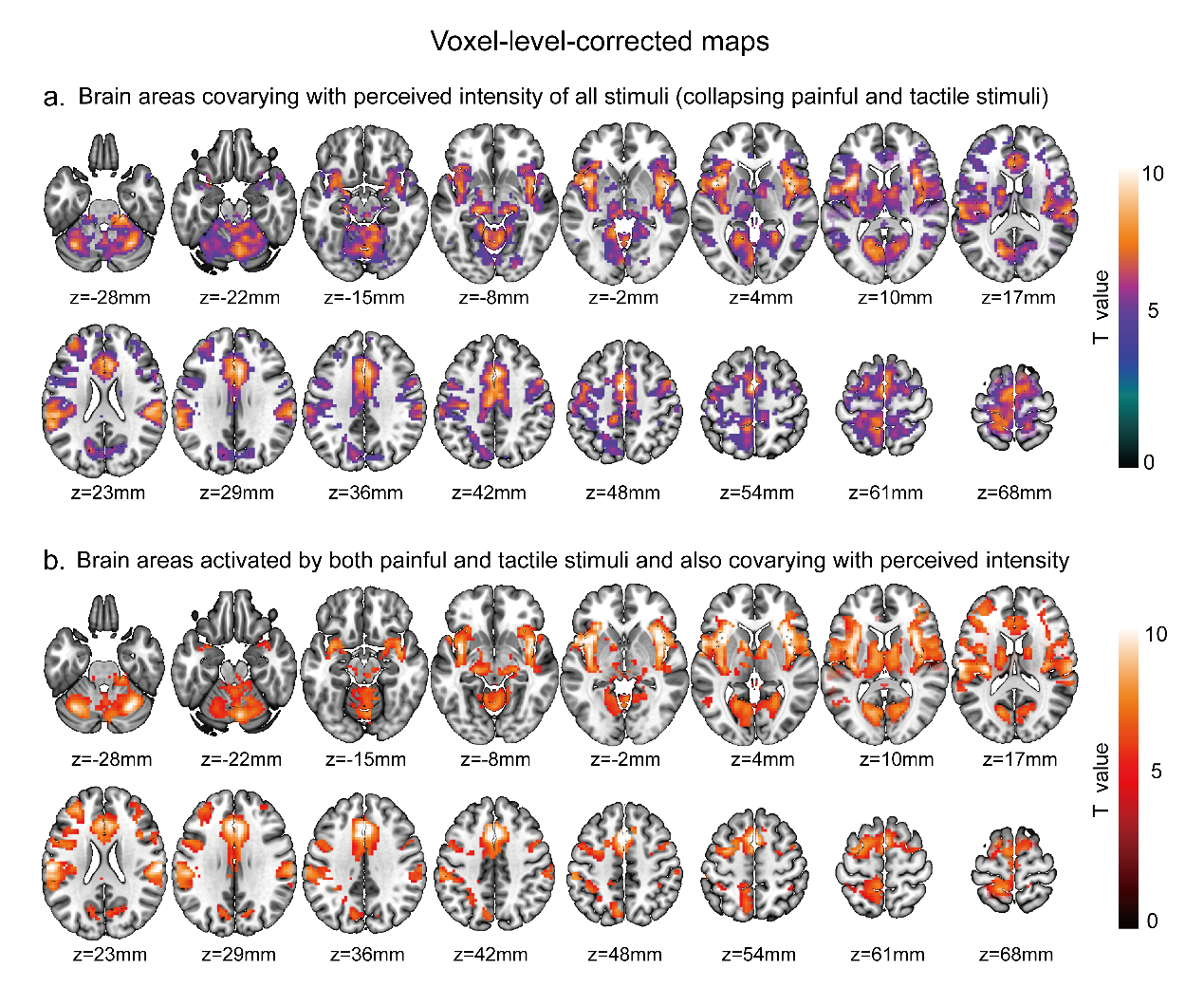


**Supplemental Figure S2.** The brain areas in which the neural activity correlated with perceived stimulus intensity regardless of stimulus modality (a) and the conjunct areas activated by both painful and tactile stimuli and at the same time correlated with the perceived stimulus intensity (b). These results were corrected at voxel level.


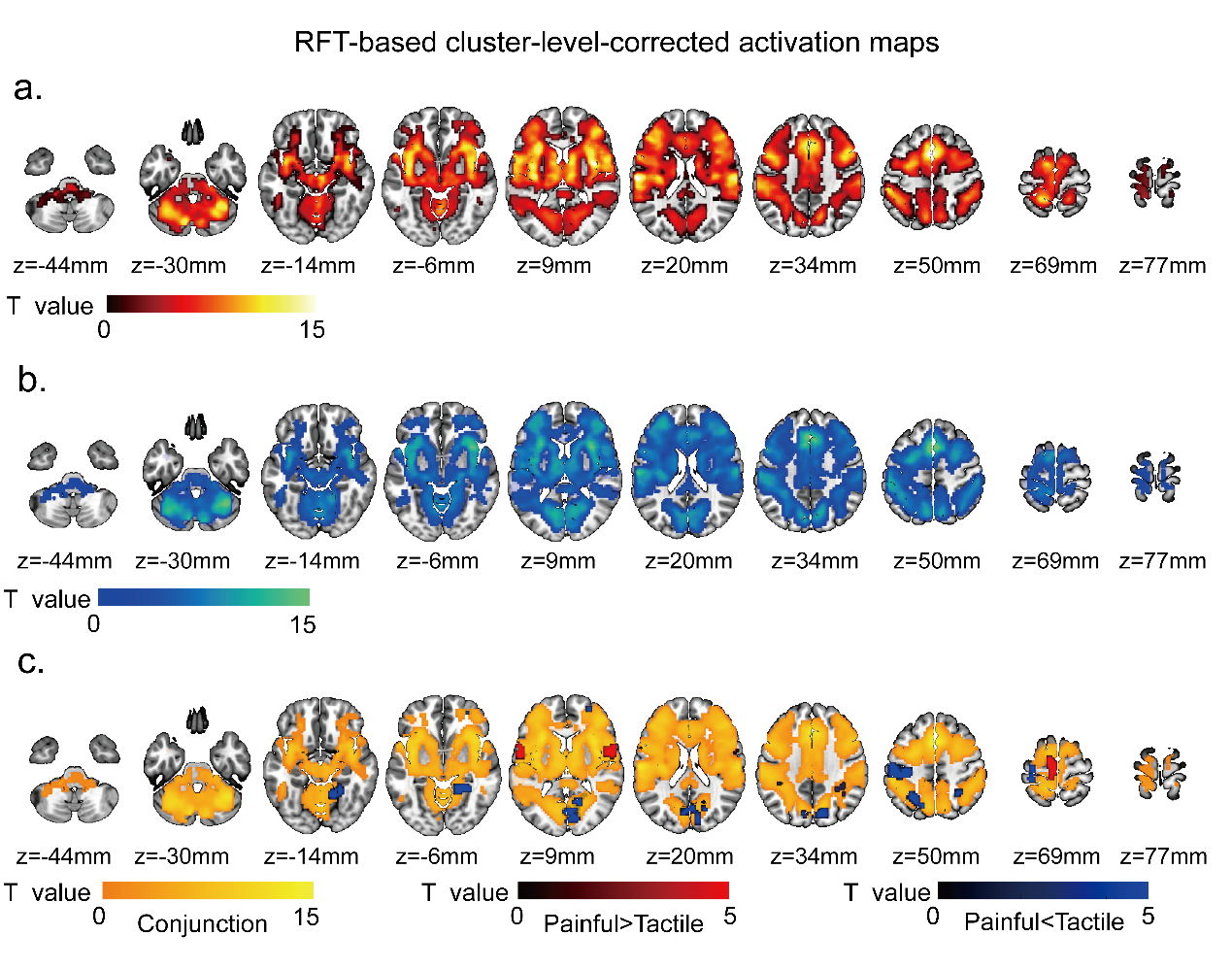


**Supplemental Figure S3.** Results of GLM analyses obtained using conventional parametric random-field-theory-based (RFT-based) family-wise-error (FWE) at cluster level (P <0.05 corrected; cluster defining threshold P <0.001) by Statistical Parametric Mapping (SPM8) software: (a) activation map by ‘intensity-matched’ painful sensation, (b) activation map by intensity-matched tactile sensation, (c) conjunct activation map (yellow areas) and the areas activated more strongly by painful stimuli than by tactile stimuli (red areas) and the areas activated more strongly by tactile stimuli than by painful stimuli (blue areas).


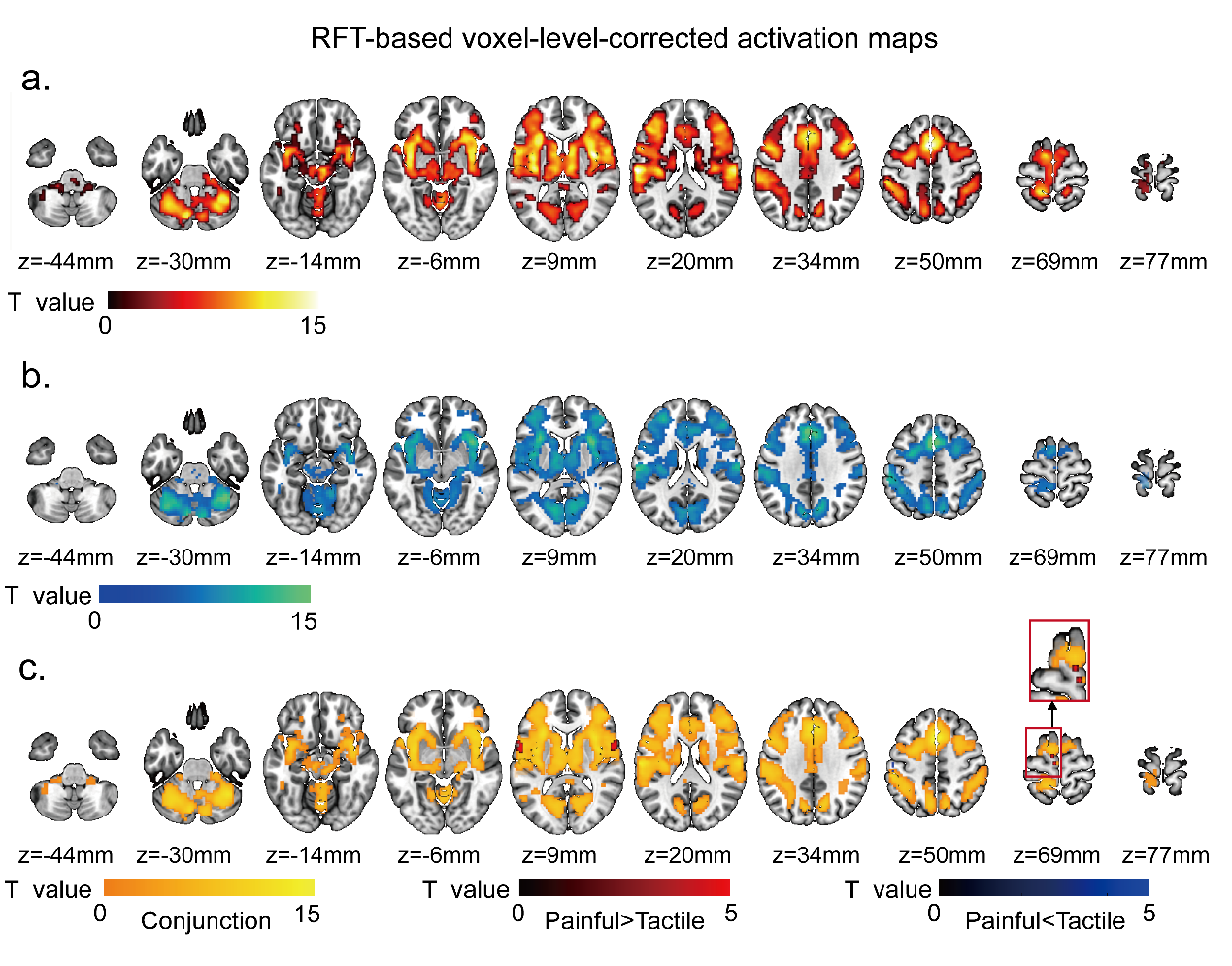


**Supplemental Figure S4.** Results of GLM analyses obtained using conventional parametric RFT-based FWE correction at voxel level (*P* *<*0.05 corrected) by SPM8 software: (a) activation map by ‘intensity-matched’ painful sensation, (b) activation map by intensity-matched tactile sensation, (c) conjunct activation map (yellow areas) and the areas activated more strongly by painful stimuli than by tactile stimuli (red areas) and the areas activated more strongly by tactile stimuli than by painful stimuli (blue areas).
